# A Review of the Principles of Radiological Assessment of Skeletal Dysplasias

**DOI:** 10.4274/jcrpe.463

**Published:** 2011-12-06

**Authors:** Yasemin Alanay, Ralph S. Lachman

**Affiliations:** 1 Pediatric Genetics Unit, Department of Pediatrics, Faculty of Medicine, Acibadem University, Istanbul, Turkey; 2 Professor Emeritus of Radiological Sciences and Pediatrics, UCLA School of Medicine, Los Angeles, California, Clinical Professor, Stanford University, Stanford CA, USA; 3 International Skeletal Dysplasia Registry, Genetics Institute, Cedars-Sinai Medical Center, Los Angeles, California, USA; +90 212 304 44 44yalanay@gmail.comAcıbadem University Faculty of Medicine, Department of Pediatrics, Pediatric Genetics Unit, Istanbul, Turkey

**Keywords:** Skeletal dysplasia, disproportionate short stature, Radiology

## Abstract

There are more than 450 well-characterized skeletal dysplasias classified primarily on the basis of clinical, radiographic, and molecular criteria. In the latest 2010 revision of the Nosology and Classification of Genetic Skeletal Disorders, an increase from 372 to 456 disorders had occurred in the four years since the classification was last revisited in 2007. These entities in total represent about 5% of children with birth defects. An accurate diagnosis of a skeletal dysplasia is still based  on detailed evaluation of clinical and radiographic [as well as chondro-osseous] findings. Regardless of the specific diagnosis, skeletal dysplasias in general share clinical and radiological findings helping  us to group them in several ways. This review aims to outline the diagnostic approach to disproportionate short stature with special emphasis on radiological findings.

**Conflict of interest:**None declared.

## INTRODUCTION

Skeletal dysplasias are disorders associated with a generalized abnormality in the skeleton. Although individually rare, the overall birth incidence is estimated to be 1/5000 live births (1). Today, there are more than 450 well-characterized skeletal dysplasias classified primarily on the basis of clinical, radiographic, and olecular criteria (2). Half a century ago, in the 1960s, individuals with disproportionate short stature were diagnosed either as achondroplasia (short-limbed dwarfism) or Morquio syndrome (short-trunked dwarfism). In time, delineation of numerous entities not fitting these two “disorders” led experts to come up with a systematic approach. The “International Nomenclature of Constitutional Diseases of Bone” group, since its first publication in 1970,  has intermittently classified these disorders  (1970-1977-1983-1992-2001-2005-2009) (3). In the 1970s, the categories were purely clinical and descriptive. This later evolved into a combination of clinical, radiological and molecular knowledge as the pathogenetic mechanisms of various entities have been revealed. In the latest 2010 revision of the Nosology and Classification of Genetic Skeletal Disorders, an increase from 372 to 456 disorders was noted in the four years since the classification was last revisited in 2007 (2,4). Of these conditions, 316 are associated with one or more of 226 different genes. This increase reflects the continuing delineation of unique phenotypes among short stature conditions, which in aggregate represent about 5% of children with birth defects (1). Some of the increase has also been driven by technological improvements in our ability to define the molecular genetic basis of these conditions, which is now known for 316 of the disorders (215 in the prior revision), with defects in 226 (140 previously) different genes. Table 1(continued 1, 2, 3, 4, 5) provides a list of main groups in the latest published classification (2). 

In daily practice however, clinicians dealing with patients with short stature may be confused with the molecular listings. It is therefore important to remember that an accurate diagnosis of a skeletal dysplasia is still based on detailed evaluation of clinical and radiographic [as well as chondro-osseous] findings. This review aims to outline the diagnostic approach to disproportionate short stature with special emphasis on radiological findings.

**Clinical Evaluation**

The accurate history regarding time of onset of short stature is essential prior to physical examination. Among the nearly 400 skeletal dysplasias, 100 or so have prenatal onset, while others may only present either as newborns or beyond 2 to 3 years of age (5). Individuals with disproportionate short stature are likely to be affected by a skeletal dysplasia. However, the abnormal proportions may not be readily recognizable. Therefore, whenever an individual presents with short stature, it is essential to measure body proportions. This should be done keeping in mind that some generalized bone mineralization abnormalities such as osteogenesis imperfects (OI), some osteosclerotic disorders, and hypophosphatasia may present with near normal proportions. 

Anthropometric measurements such as upper/lower segment (U/L) ratio, sitting height, and arm span are routinely measured when a patient with short stature is evaluated. Sitting height is the measurement of head and trunk, and may be difficult to measure accurately due to the need of special equipment. The lower segment, however, is easier to measure (from symphysis pubis towards the floor medially to the heel). The upper segment can then be 

easily calculated by subtracting the lower segment from total height. Upper and lower segment measurements can be made in a standing or supine position. The mentioned ratios change with age. U/L ratio is 1.7 in the newborn; approximately 1.0 between ages 2-8 years; 0.95 as an adult. A short statured patient with short trunk will have decreased a U/L ratio, while an individual with normal trunk and relatively short limbs will have an increased U/L ratio (6).

Clinical evaluation also includes description of the limb involvement. Depending on the primarily involved segment of the limb, the condition can be described as rhizomelic (humerus and femur), mesomelic (radius, ulna, tibia and fibula) and acromelic (hands and feet). These descriptions help in differential diagnosis.  It is noteworthy that a careful examination by an experienced clinical geneticist can sometimes narrow the list of dysmorphological entities to be considered even before the skeletal radiographs are analyzed (7).

Other clinical assessments such as immunological /hematological data as well as hair quality, cleft palate, eye abnormalities (myopia) and even internal organ abnormalities (cystic kidneys, hepatosplenomegaly) are important in skeletal dysplasia evaluation.

After obtaining a thorough family history, constructing a detailed pedigree and performing clinical examination, radiological assessment is likely to close the case in most skeletal dysplasias as many have distinctive radiological features in growing bones.

**Radiological Assessment **

Before giving details of the stepwise radiographic analysis for skeletal dysplasias; we would like to emphasize that a complete “ genetic skeletal survey” is not necessary in patients with proportionate short stature, in which the differential diagnosis consists of constitutional delay, familial short stature, a small group of endocrinopathies and some dysmorphic syndromes. Their initial imaging assessment may warrant a left hand and wrist radiograph for bone age determination. This will protect children from unnecessary radiation exposure. 

The “genetic skeletal survey” should include anteroposterior (AP), and lateral views of the skull, AP and lateral views of the entire spine, and AP views of the pelvis and all four extremities, with separate AP views of the hands and feet [A lateral view of the knee can be helpful to diagnose a recessive form of multiple epiphyseal dysplasia (MED) associated with multilayered patella] (7). In adult patients, it is mandatory to try to obtain prepubertal skeletal radiographs. Once the epiphyses have fused to the metaphyses, diagnosis may be very difficult. After obtaining the radiographs, a three-step assessment will be helpful in trying to make a specific diagnosis.

**Step I (Assessment of Disproportion):** An assessment of disproportion similar to the one made clinically is repeated looking at the radiographs. A quick look at the spine will readily help decide if there is platyspondyly leading to short-trunked disproportion. Similarly, looking at the extremities may help defining rhizomelia, mesomelia, and acromelia. It should be noted that these descriptive terms of limb segments may be more correct radiologically as the clinical visualization is accentuated by skin folds or other tissues rather than the length of the underlying bone. Rhizomelic chondrodysplasia punctata (CDP) is a good example of a rhizomelic skeletal dysplasia diagnosed with the additional radiological findings of  punctate calcifications (stippling) and coronal clefted vertebrae (Figure 1). Mesomelia alone will suggest a long heterogeneous differential diagnosis list of mesomelic dysplasias. Presence of acromelia is important to recognize, as it may be an isolated finding. Presence of isolated acromelia may suggest skeletal dysplasias such as acromicric dysplasia,  acrodysostosis, geleophysic dysplasia or nonskeletal dysplasias such as the brachydactylies. Brachydactyly type E, characterized by a short fourth metacarpal bone may support clinical or laboratory findings in Turner syndrome and pseudohypoparathyroidism, respectively.  The absence of proportional acromelic shortening is also very important to remember in spondyloepiphyseal dysplasia congenita (and most forms of type II collagenopathies) (7).

**Figure 3a:**
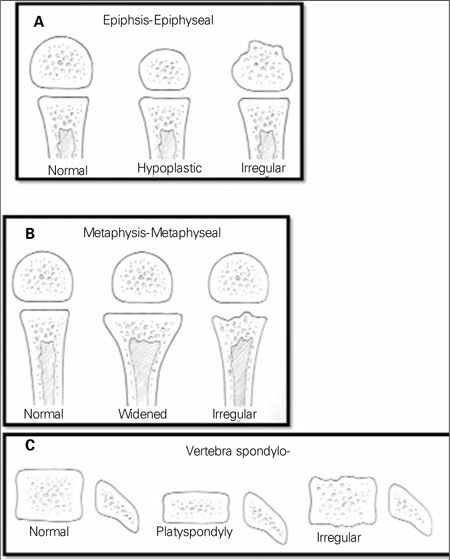
a,b,c. Radiographic manifestations of the dysplasias

**Figure 4:**
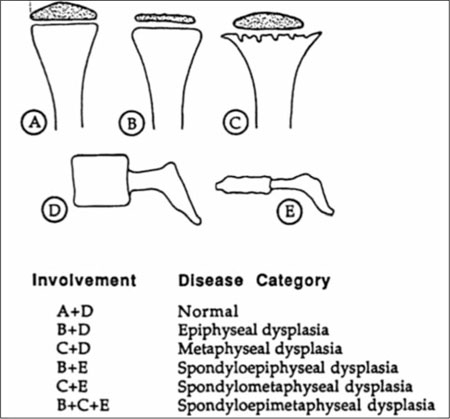
Radiographic abnormalities helpful in classification of skeletal Dysplasias

**Figure 5a and 5b:**
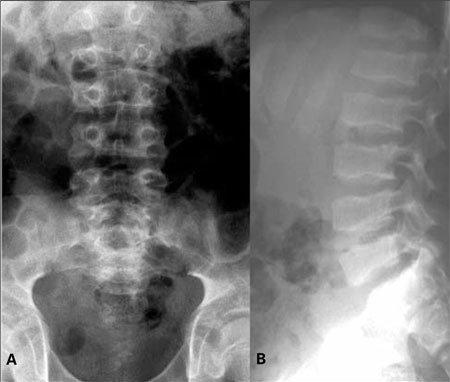
a,b. Brachyolmia. Note platyspondyly and overfaced pedicles

**Step II (Assessment of Epiphyseal/Metaphyseal/ Diaphyseal Ossification):** Abnormal development of epiphyses, metaphyses, and diaphyses has given rise to the original nomenclature using those site names (Figure 2). An overall look at the radiological survey will suggest epiphyseal dysplasias by the presence of very small (delayed ossification) and/or irregularly ossified epiphyses (Figure 3a). If the metaphyses are widened, flared, and/or irregular, the diagnosis of a form of metaphyseal dysplasia is established (Figure 3b, 3c and Figure 4). Diaphyseal dysplasia is present when there is diaphyseal widening and/or cortical thickening or marrow space expansion or restriction. Isolated vertebral involvement without changes in the growth plate region in a patient with short-trunked short stature should suggest brachyolmia (Figure 5a and 5b). Figure 3 helps to combine the aforementioned skeletal involvement, such as forms of spondyloepiphyseal dysplasia and the group of spondylo-epi-(meta)-physeal dysplasias [SE(M)Ds]. Fractures can be seen in all types of OI (Figure 6), osteosclerotic disorders including osteopetrosis (Figure 7) and severe hypophosphatasia (Figure 8) (7).

Following the evaluation of limb segments and the epiphyseal growth plate, focus on all the skeletal structures available in the genetic skeletal survey is mandatory to recognize a well-described skeletal dysplasia from a previous broad categorization into a specific group. This precise evaluation will include a search for pathognomonic findings, such as snail-shaped iliac bones of Schneckenbecken dysplasia (Figure 9), “lacy” appearance of iliac crest in Dyygve-Melchior-Clausen syndrome (Figure 10), and loss of mandibular angle accompanied by wormian bones and acroosteolysis in pycnodysostosis (Figure 11 a,b,c) (7).

**Step III (Differentiation of Normal Variants from Pathological Abnormalities):** This last step requires experience in the field of pediatric radiology. It essentially involves recognition of normal variation from pathological abnormalities in the growing skeleton. Every portion of every bony structure should be looked at in an effort to combine the clinical, often dysmorphic findings previously noted in evaluation of the patient. Pathognomonic findings help to narrow the group of differential diagnosis leading to a specific entity. 

At this point, having had a thorough clinical and radiographic assessment, even a simple radiographic grouping can be helpful to the clinician for the establishment of clinical care and follow-up. Table 2(continued 1, 2, 3, 4) provides a list of the grouping mentioned with common specific entities to consider (7). If a specific diagnosis cannot be made, it is expedient to send the case to a local expert, or an expert group in Skeletal Dysplasias such as the International Skeletal Dysplasia Registry at Cedars Sinai MC [www.csmc.edu/skeletaldysplasia].

**(Figure 1):**
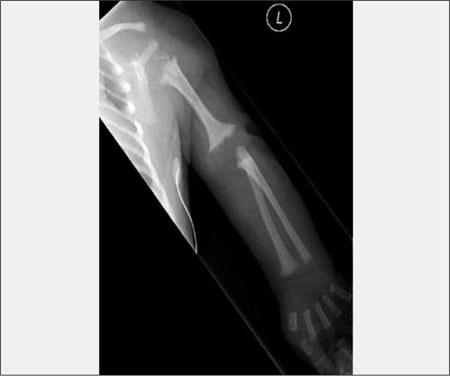
Rhizomelic chondrodysplasia punctata. Note shortness of humerus

**(Figure 2):**
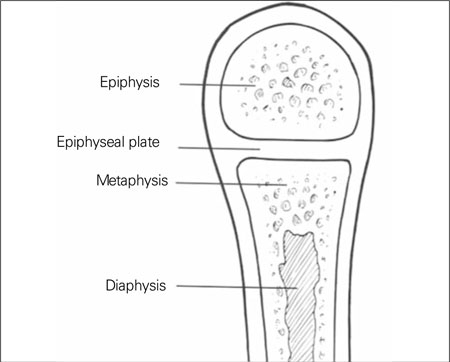
Key areas of the growing bone

**(Figure 6):**
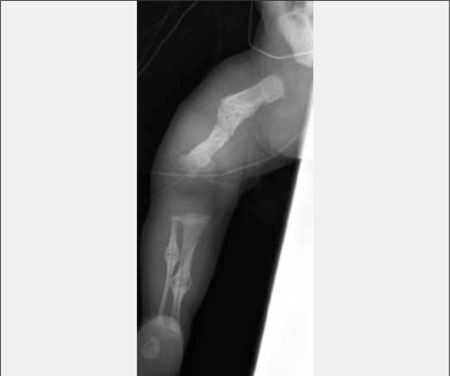
Fractures in Osteogenesis Imperfecta

**(Figure 7):**
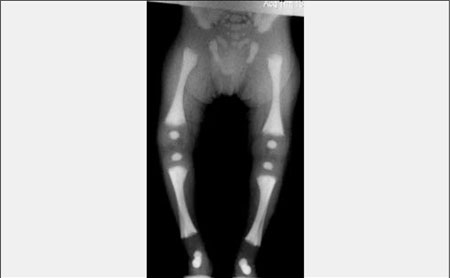
Osteopetrosis, generalized osteosclerosis

**(Figure 8):**
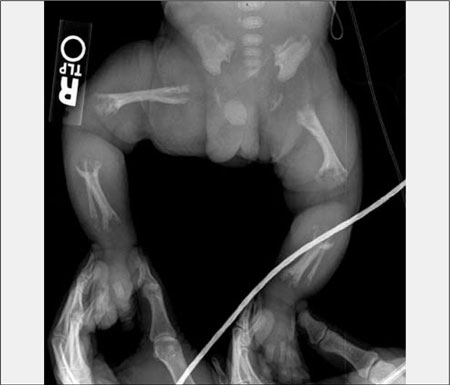
Infantile hypophosphatasia

**(Figure 9):**
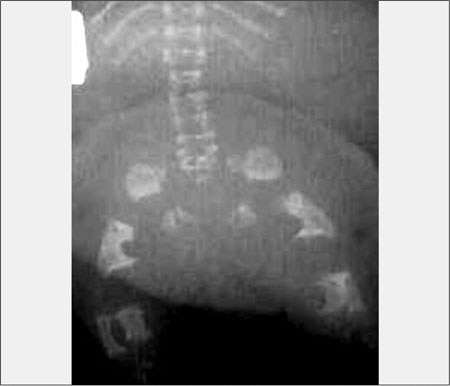
Schneckenbecken dysplasia. Note severe platyspondyly, thin ribs and snail-shaped iliac bones

**(Figure 10):**
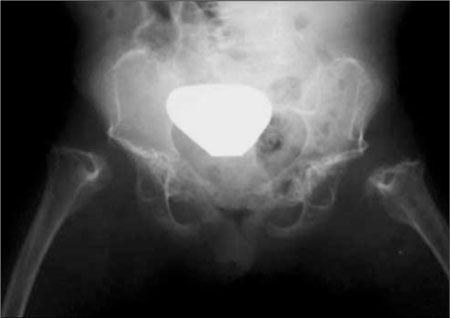
Dyygve-Melchior-Clausen syndrome. Note “lacy” iliac crest

**(Figure 11 a,b,c):**
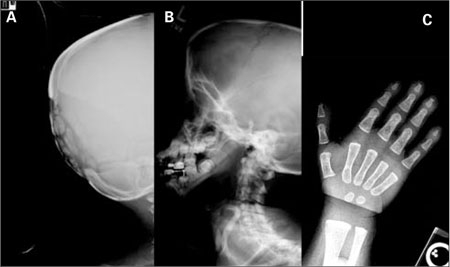
a,b,c. Pycnodysostosis. Loss of mandibular angle with wormian bones, large fontanelle and acroosteolysis in distal phalanges of hand

**Table 1:**
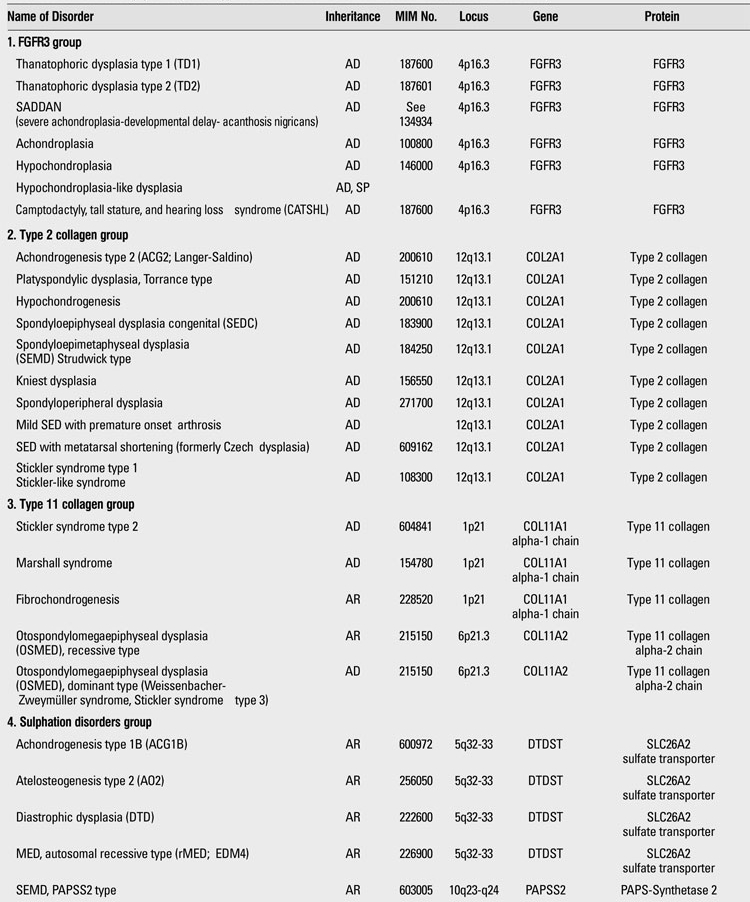
Osteochondrodysplasias (Nosology and Classification of Genetic Skeletal Disorders: 2010 Revision

**1:**
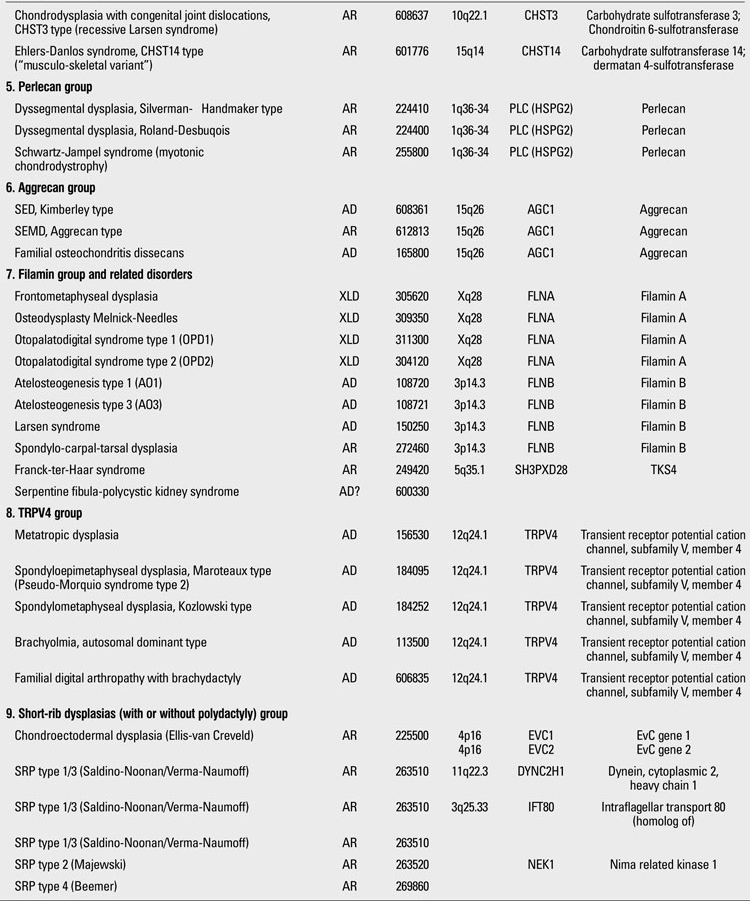
Osteochondrodysplasias (Nosology and Classification of Genetic Skeletal Disorders: 2010 Revision

**2:**
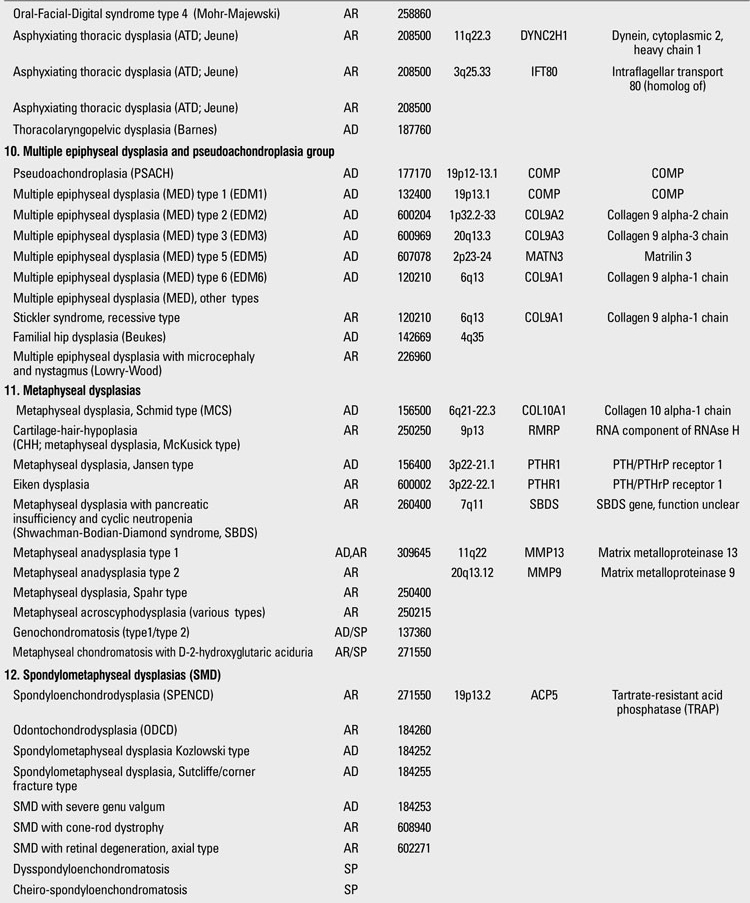
Osteochondrodysplasias (Nosology and Classification of Genetic Skeletal Disorders: 2010 Revision

**3:**
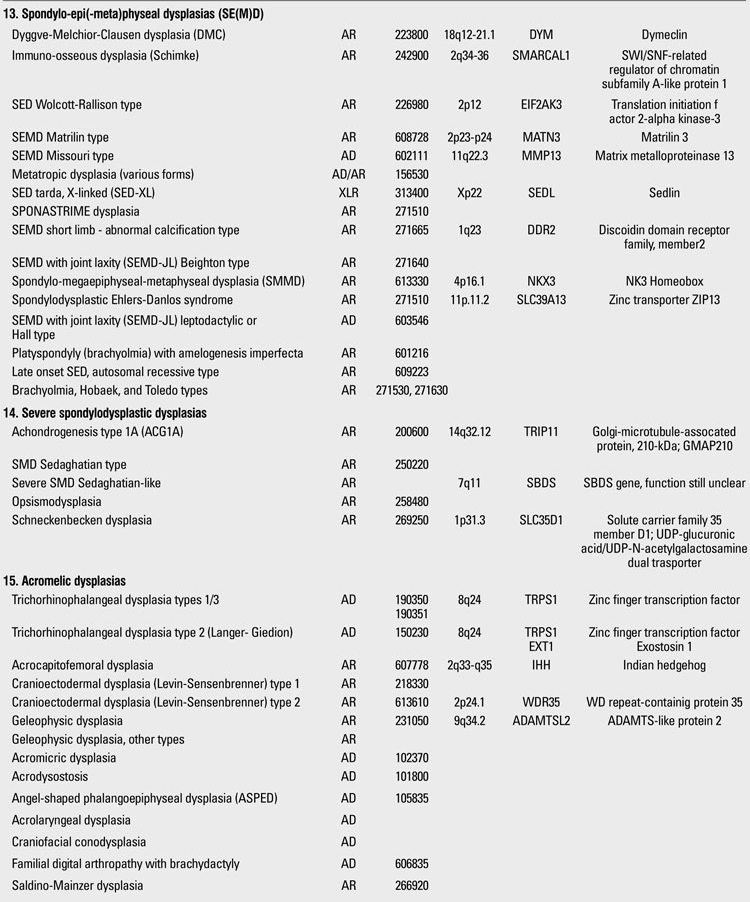
Osteochondrodysplasias (Nosology and Classification of Genetic Skeletal Disorders: 2010 Revision

**4:**
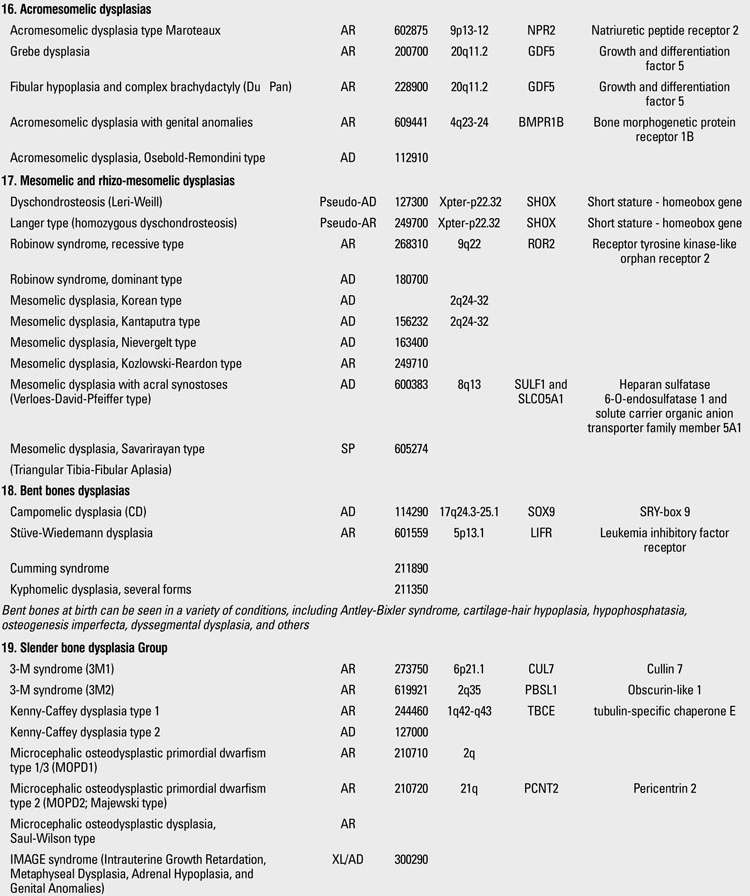
Osteochondrodysplasias (Nosology and Classification of Genetic Skeletal Disorders: 2010 Revision

**5:**
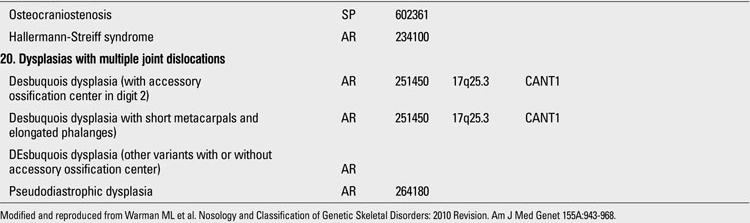
Osteochondrodysplasias (Nosology and Classification of Genetic Skeletal Disorders: 2010 Revision

**Table 2:**
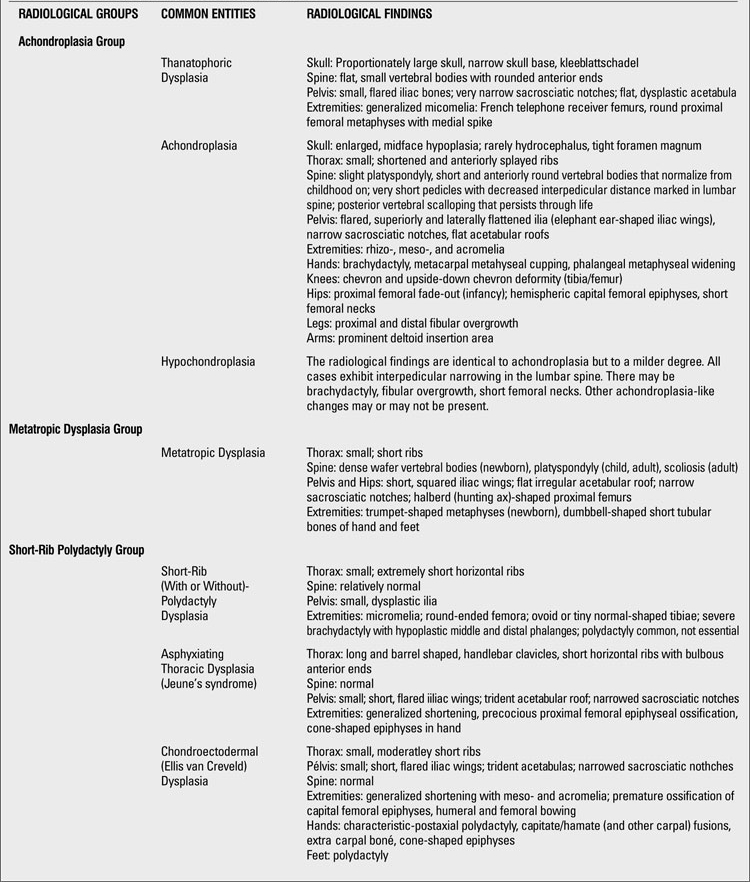
Clues for radiographic diagnosis of skeletal dysplasias

**1:**
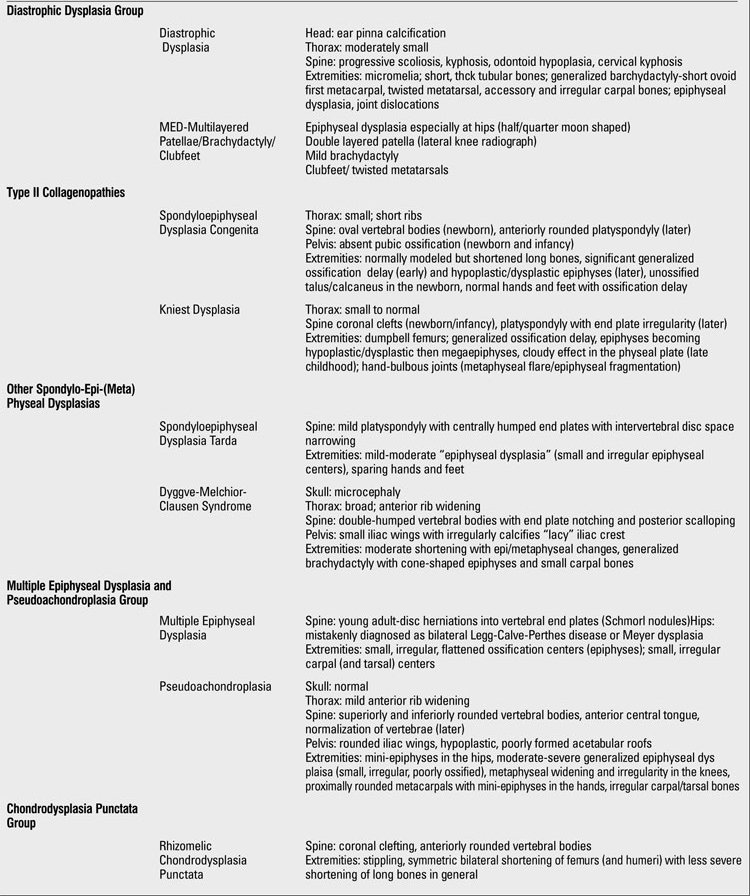
Clues for radiographic diagnosis of skeletal dysplasias

**2:**
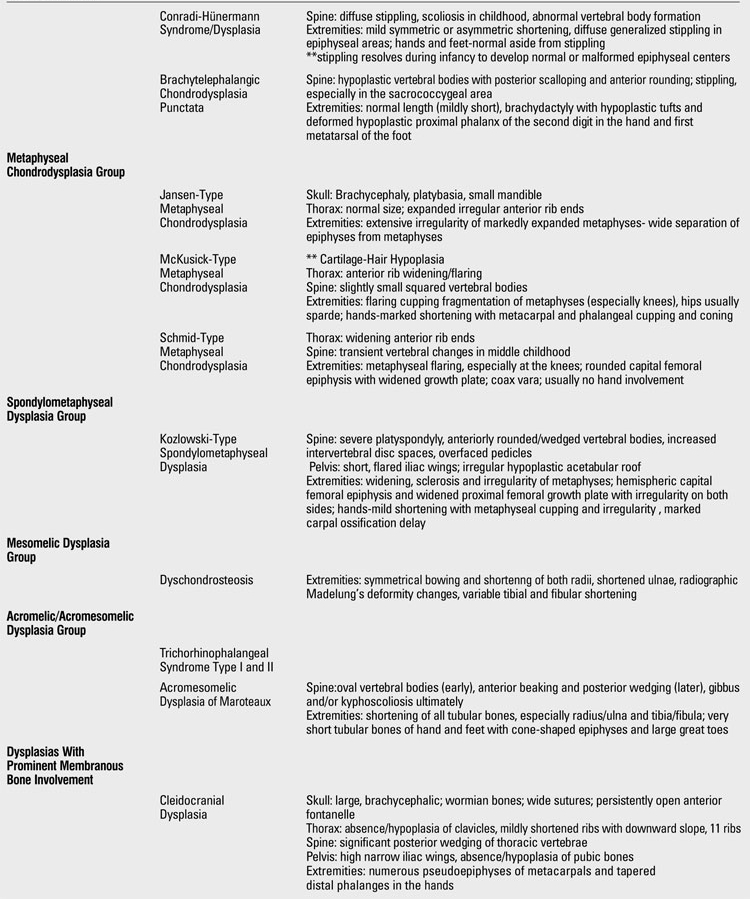
Clues for radiographic diagnosis of skeletal dysplasias

**3:**
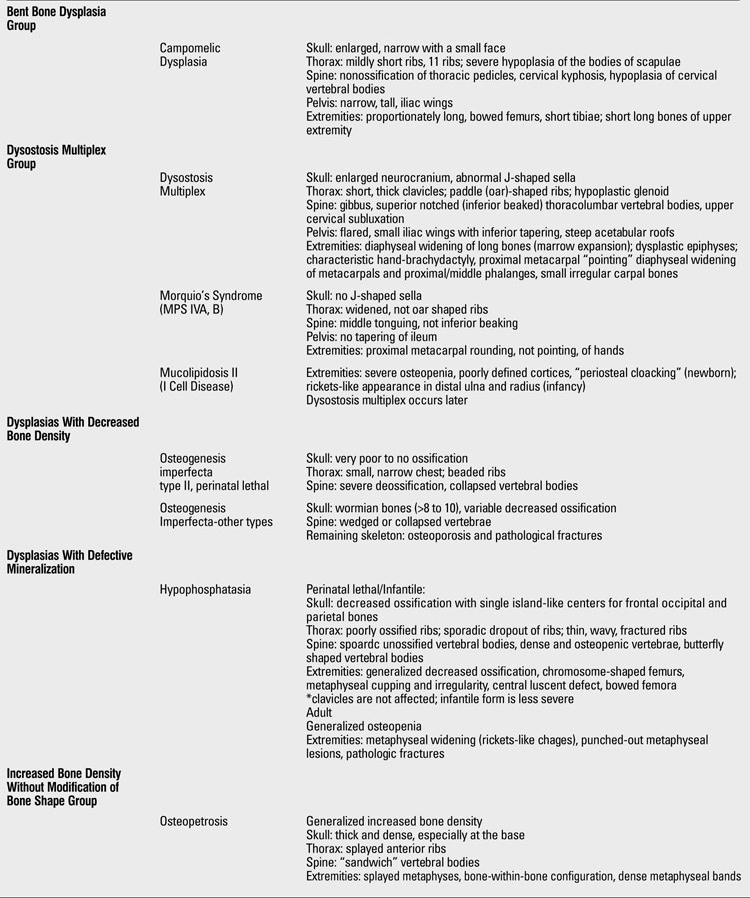
Clues for radiographic diagnosis of skeletal dysplasias

**4:**
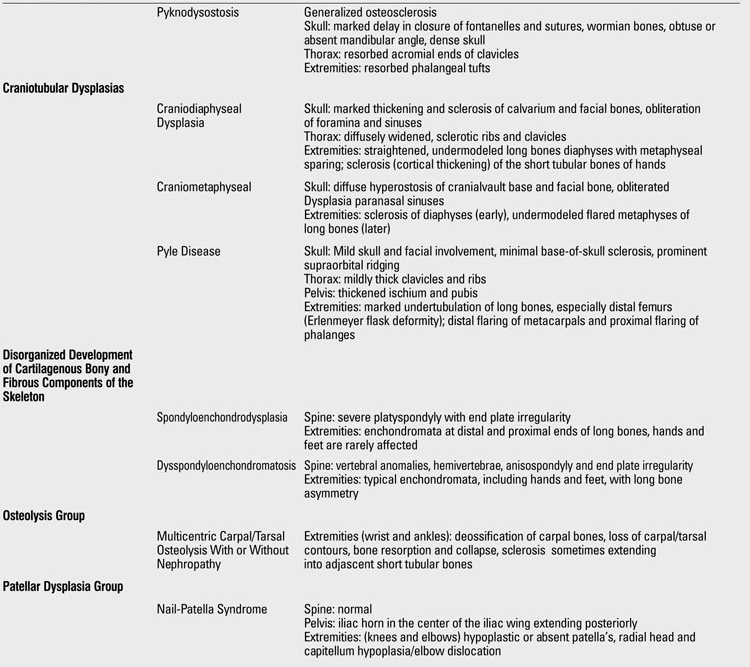
Clues for radiographic diagnosis of skeletal dysplasias

## CONCLUSION

The complete group of osteochondrodysplasias, although individually rare, is an important group of disorders for healthcare providers who deal with individuals with short stature. These individuals present with significant morbidities due to destruction of bone and cartilage caused by defects in linear growth, bone modeling and regeneration.  Regardless of the specific diagnosis, skeletal dysplasias in general share clinical and radiological findings helping us to group them in several ways. In this review, we aimed to focus on the radiological aspect of assessment of skeletal dysplasias. We also included an outline of the basic clinical approach to an individual with a suspected skeletal dysplasia. The recent advances in the field of molecular pathogenetic mechanisms underlying skeletal dysplasias are beyond the scope of this review. However, we would like to emphasize that accurate clinical, radiological and finally molecular diagnosis of skeletal dysplasias is more important than ever in this era of up-to-date genetic counseling, prenatal, preimplantation genetic diagnoses and hopefully, molecularly targeted therapeutics in the future.

**Acknowledgement**

RSL, is supported by NIH GRANT# HD 22657.   
